# Effect of Providing Multiple Micronutrients in Powder through Primary Healthcare on Anemia in Young Brazilian Children: A Multicentre Pragmatic Controlled Trial

**DOI:** 10.1371/journal.pone.0151097

**Published:** 2016-03-14

**Authors:** Marly A. Cardoso, Rosangela A. Augusto, Gisele A. Bortolini, Cristieli S. M. Oliveira, Daniela C. Tietzman, Leopoldina A. S. Sequeira, Maria Claret C. M. Hadler, Maria do Rosario G. Peixoto, Pascoal T. Muniz, Márcia R. Vitolo, Pedro I. C. Lira, Patrícia C. Jaime, ENFAC Working Group

**Affiliations:** 1 Department of Nutrition, School of Public Health, University of São Paulo, São Paulo, Brazil; 2 Coordenação Geral de Alimentação e Nutrição (CGAN), Ministry of Health, Brasília, Brazil; 3 Department of Health Sciences, Federal University of Acre, Rio Branco, Brazil; 4 Universidade Federal de Ciências da Saúde de Porto Alegre (UFCSPA), Porto Alegre, Brazil; 5 Department of Nutrition, Universidade Federal de Pernambuco, Recife, Brazil; 6 School of Nutrition, Federal University of Goiás, Goiânia, Brazil; TNO, NETHERLANDS

## Abstract

**Background:**

Multiple micronutrients in powder (MNP) are recommended by WHO to prevent anemia in young children. However, evidences for its effectiveness in different populations and improvements in other outcomes (e.g. linear growth and vitamin A deficiency) are scarce.

**Methods:**

A multicentre pragmatic controlled trial was carried out in primary health centres. At study baseline, a control group (CG) of children aged 10- to 14 months (*n* = 521) was recruited in the routine healthcare for assessing anemia, anthropometric and micronutrient status. At the same time, an intervention group (IG) of infants aged 6- to 8 months (*n* = 462) was recruited to receive MNP daily in complementary feeding over a period of 60 days. Both study groups were compared when the IG infants reached the age of the CG children at enrolment.

**Results:**

In CG, the prevalence of anemia [hemoglobin (Hb) < 110 g/L], iron deficiency (ID, plasma ferritin < 12 μg/L or TfR > 8.3 mg/L), and vitamin A deficiency (VAD, serum retinol < 0.70μmol/L) were 23.1%, 37.4%, and 17.4%, respectively. Four to six months after enrolment, when the IG participants had the same age of the controls at the time of testing, the prevalence of anemia, ID and VAD in IG were 14.3%, 30.1% and 7.9%, respectively. Adjusting for city, health centre, maternal education, and age, IG children had a lower likelihood of anemia and VAD [Prevalence Ratio (95% CI) = 0.63 (0.45, 0.88) and 0.45 (0.29, 0.69), respectively] when compared with CG children. The adjusted mean distributions of Hb and length-for-age Z-scores improved by 2 SE in the IG compared to CG children.

**Conclusions:**

MNP effectively reduced anemia and improved growth and micronutrient status among young Brazilian children.

**Trial Registration:**

Registro Brasileiro de Ensaios Clinicos RBR-5ktv6b

## Introduction

Anemia in children is a major public health problem in both developed and developing countries, affecting an estimated 293 million preschool-aged children [1]. In recent decades, numerous strategies for anemia prevention and control have been adopted around the world, but few have successfully reduced its prevalence. As global estimates on micronutrient deficiencies are not available, prevalence of anemia in preschool children is often used as an outcome for micronutrient deficiencies [2]. The WHO estimates the prevalence of iron deficiency in 2.5 times higher than the prevalence of anemia in children under 2 years [2–3]. Although iron deficiency resulting from low intake and low bioavailability of dietary iron remains the main cause of anemia, especially in poor areas, many other factors play a role, including genetics, infections, and other micronutrients deficiencies, including vitamin A and folate [2–3].

A review of studies carried out in Brazil from 1996–2007 reported a 53% prevalence of anemia among children aged 6–59 months, with the highest prevalence among children under 24 months of age [4–5]. The role of multiple micronutrient deficiencies in the etiology of anemia has been considered in recent studies [6–7]. Brazil has adopted three strategies to prevent and control anemia: nutrition education, mandatory fortification of flour, and iron supplementation in children under 18 months and in pregnant and lactating women. Nutrition education has been implemented in primary health care [8]. Fortification of wheat and corn flours with iron and folic acid was introduced in June 2004, with 4.2 mg of iron and 150 mg of folic acid added per 100 g of flour. The fortification of flour with folic acid has produced positive results in the U.S., Canada and Chile, reducing the incidence of neural tube defects [9]. However, the effectiveness of iron fortification of flour in preventing childhood anemia appears to be low. In Brazil, a study assessing the impact of iron-fortified flour on hemoglobin concentrations among preschool children did not find a statistically significant effect [10]. Given the amount of flour typically present in complementary feeding, infants should not be considered the target group for whom flour fortification has a positive impact.

The transition to complementary foods as children begin to consume household diets is associated with insufficient iron intake, combined in many cases with insufficient or borderline dietary intake of other micronutrients [11–12]. Thus, in 2011, a WHO technical publication examined strategies to prevent and control anemia in children aged 6–23 months, suggesting the use of multiple micronutrients in powder (MNP) as a home-based strategy for health promotion [1]. This approach uses sachets containing a mixture of powdered vitamins and minerals that can be easily mixed with semi-solid foods. Iron in the powder (as ferrous fumarate) is encapsulated in a lipid layer to prevent its interaction with foods, which can alter food texture [13]. However, most of the evidence relating to MNP reported in Cochrane systematic reviews derives from African and Asian studies [14–15]. Those studies also showed that home fortification is effective in preventing iron deficiency (ID) and iron deficiency anaemia (IDA). However, the effect of home fortification of complementary feeding on plasma levels of vitamin A is unconvincing. There are also no clear effects of this strategy on growth.

The present study aimed to evaluate the effectiveness of home fortification of complementary feeding with MNP delivered through the primary healthcare provider in preventing anemia and improving growth and micronutrient status among young Brazilian children.

## Materials and Methods

### Study population and design

The *Estudo Nacional de Fortificação caseira da Alimentação Complementar* (ENFAC) is a pragmatic controlled clinical trial designed to assess the impact of MNP on anemia of infants seen at primary healthcare practices in Brazil. A complete list of the ENFAC investigators is listed in the Acknowledgments. This study was registered at www.ensaiosclinicos.gov.br as RBR-5ktv6b (S1 and S2 Texts). For this study, 24 large primary health centers (PHC) in four cities from four different regions in Brazil were selected, based on the largest enrolment in each city: Goiânia, 5 PHC; Olinda, 9 PHC; Rio Branco, 6 PHC; Porto Alegre, 4 PHC. Goiania, the state capital of Goiás, located in the Midwestern region of the country, with 1,301,892 inhabitants (2010 Census) and a Human Development Index (HDI) of 0.832. Olinda, a city of Recife metropolitan region of the state of Pernambuco, located in the northeast of the country, with a population of 375,559 inhabitants and an HDI of 0.801. Rio Branco, capital of Acre state, is located in northern Brazil, with 335,796 inhabitants, nearly half the state's population and HDI of 0.754. Porto Alegre, the state capital of Rio Grande do Sul, had 1,409,351 inhabitants and IDH of 0,865 [16]. From June 2012 to April 2013, the mothers or guardians of children aged 10–14 months receiving routine pediatric care were invited to participate in the study as the control group (CG). At the same time, an intervention group (IG) of infants aged 6–8 months was recruited through the same health centers to receive home fortification with MNP given once daily in complementary feeding over a period of 60 days, as per WHO guidelines [2]. Because of ethical constraints of collecting blood samples from 6–8 month-infants in the routine primary healthcare and keeping possible anemic children without the standard treatment with iron supplementation, we did not collect blood samples from IG infants before the intervention. The IG infants were assigned to the intervention for a follow-up period of four to six months after enrolment. Thus, both study groups were compared when the IG infants reached the age of the CG children at enrolment. Laboratory results were provided to the caregivers or community health workers for further follow-up and treatment when necessary.

A sample size of at least 105 children in each IC and CG groups was estimated to detect an increase in mean blood hemoglobin (Hb) concentration of 6 g/L (SD = 12g/L) in the intervention group with a power of 0.95 and an α two-tailed level of 0.05 [17]. The sample size in each study city was increased to 135 to account for dropouts in the IG, expecting at least a total of 1080 children (540 in each IC and CG groups) at recruitment. Overall, 1225 children were assessed for eligibility from June 2012 to December 2012. The eligibility criteria for participation were as follows: 1) parental approval to participate in the study and 2) not currently receiving treatment for anemia. Exclusion criteria included premature birth (<37 weeks’ gestation); twins; reported cases of HIV infection, malaria, tuberculosis or genetic Hb disorders; and fever (>39°C) on the day of blood sampling.

Written informed consent was obtained from each child’s primary caregiver.

The Human Ethical Review Board of the School of Public Health, University of São Paulo, Brazil, and the institutional review boards of Federal University of Rio Branco, The Universidade Federal de Ciências da Saúde de Porto Alegre, The Universidade Federal de Pernambuco, and The Federal University of Goiás approved the research protocol.

### The intervention

The nutrient composition of the MNP is shown in Table 1. The MNP sachets (dose = 1 sachet/day) were to be mixed with the infant’s meal immediately before serving. Health workers were invited to a 1-hour learning session provided by the research team before MNP distribution. As previously described for communications designed to change behavior [18], the learning session covered why children need iron, the benefits of MNP in reducing childhood anemia and micronutrient deficiencies, and the appropriate use of MNP. The research team provided technical assistance to health workers, with materials to help develop the MNP intervention and to integrated it with the existing nutrition program. Health care workers provided a 2-month supply (60 sachets per child) of MNP to caregivers.

**Table 1:** Nutrient composition of the MNP used in the ENFAC Study^a^.

Nutrient	Amount/1g
Iron (ferrous fumarate), *mg*	10
Zinc (gluconate), *mg*	4.1
Folic acid, *μg*	150
Vitamin A (RE), *μg*	400
Viamin C, *mg*	30
Vitamina D_3_, μg	5
Vitamin E (TE), *mg*	5
Vitamin B_1_, *mg*	0.5
Vitamin B_2_, *mg*	0.5
Vitamin B_6_, *mg*	0.5
Vitamin B_12_, *μg*	0.9
Niacin, *mg*	6
Cupper, *mg*	0.56
Iodine, *μg*	90
Selenium, *μg*	17

^a^ Individual serving of 1g/sachet to be mixed with semi-solid food daily for 60 days. MNP, multiple micronutrient powder; ENFAC Study, *Estudo Nacional de Fortificação caseira da Alimentação Complementar*.

### Data collection

The CG was enrolled at 10–14 months of age. On enrolment, a questionnaire was administered through a face-to-face interview with the mothers or guardians of all participants. The interview, conducted by trained fieldworkers, addressed the following topics: demographic characteristics (child’s sex, age, and race/ethnicity, classified as white, black, “pardo” [brown], yellow, or indigenous, according to skin color, as used in the Brazilian census); maternal education; reproductive health variables (maternal age, birth weight); and morbidity (diarrhea, wheezing, or fever up to 15 days prior to the interview; episodes of malaria in the past 12 months). Length was measured by trained research assistants according to standardized procedures [19] using portable infant measuring boards (model ES-2000, Sanny, Los Angeles, USA). Each measurement was repeated twice and the mean value calculated. Z-scores for length/height-for-age (HAZ) were calculated according to the WHO child growth standards [20]. Stunting was defined as HAZ of <-2.0 [19]. The IG infants were enrolled at 6–8 months of age to receive the intervention. Four to six months after enrolment, research interviewers performed the same data collection procedures described above for the CG children, with additional questions on MNP consumption and a count of unused sachets from each household at the end of the trial.

### Laboratory methods

A fasting (≥ 3 hours) venous blood sample (around 5 mL) was collected in the morning on a day scheduled with caregivers (at enrolment for the CG and 4–6 months after enrolment for the IG children). Because limited sample volumes were available during some difficult blood collection, not all laboratory analyses were performed for all children. At the field laboratory, whole blood aliquots collected in EDTA-containing vacuum tubes were used to measure Hb concentrations on portable hemoglobinometers (Hb301; HemoCue^®^, Angelholm, Sweden) by trained nurses following recommended standardization procedures [21]. A separate blood sample was protected from light and centrifuged within 1 hour of collection; serum and plasma samples were frozen at -20°C before being shipped to São Paulo on dry ice and maintained at -70°C until further analysis. In São Paulo, plasma ferritin (PF) and soluble transferrin receptor (sTfR) concentrations were measured using commercially available enzyme immunoassays (Ramco, Houston, TX); C-reactive protein (CRP) > 5mg/L and α-1-acid glycoprotein (AGP) > 1g/L were measured as acute and chronic inflammation [22], respectively, using an IMMAGE Immunochemistry System (Beckman Coulter, Brea, CA, USA). Anemia, ID, and IDA were defined according to Hb, PF, and sTfR as follows: anemia was defined as Hb concentration <110 g/L. ID was defined as PF concentrations <12 μg/L or sTfR concentrations >8.3 mg/L. IDA was defined as ID occurring in anemic children. Serum folate concentrations were measured using commercial fluoroimmunoassays (Perkin Elmer, Wallac Oy, Turku, Finland); values <10 nmol/L were considered diagnostic of folate deficiency [23]. Serum concentrations of β-carotene, retinol and vitamin E were measured by HPLC methods (HP-1100 HPLC system, Hewlett Packard, Palo Alto, California, USA) as previously described [24]; serum retinol concentration <0.70 μmol/L was used to indicate vitamin A deficiency [25]. Frozen samples were analyzed within 6 months of collection. The laboratory assayed internal and external blinded quality control specimens in each run. Based on the control specimens, the accuracy and interassay coefficients of variation for these analyses were within 7%.

### Outcome variables

The primary outcome measure was the difference in mean Hb between the IG using home fortification with MNP and the CG. Secondary outcome measures were the prevalence of anemia, iron and vitamin A deficiencies, and mean HAZ. Both study groups were compared when the IG infants reached similar age of the CG children at enrolment.

### Statistical analysis

The intervention effects were assessed with the use of intention-to-treat analysis as we did not consider the compliance for the use of MNP into the analysis. Median values and IQR were calculated for micronutrient concentrations, dietary intake, and continuous covariates according to outcome status. Pearson χ^2^ and Student **t** tests were used to examine differences in proportions and in continuous variables between groups, respectively.

We used mixed-effects Poisson regression models with robust variance to estimate adjusted values for the prevalence ratios (PR) for the outcome variables. Because of the hierarchical nature of the data, we used multilevel Poisson regression analyses with an extra Poisson variation to estimate the level-1 (individual level) variance. The IG was compared with the CG while controlling for city, primary health center, maternal schooling, and child age. In subsequent analyses, adjusted individual level data for Hb and HAZ were used to compare the distributions between study groups (using *kdensity* and *normalden* procedures in Stata 13.0 to illustrate the true underlying density for continuous random variables). Missing data (less than 10%) were included in the multiple models by creating missing-value categories. Statistical significance was set at 0.05, and PR with 95% CI are presented. All *P* values were derived from two-sided statistical tests. All analyses were done in STATA 13.0 (Statacorp, College Station, Texas, USA).

## Results

**Fig 1:**
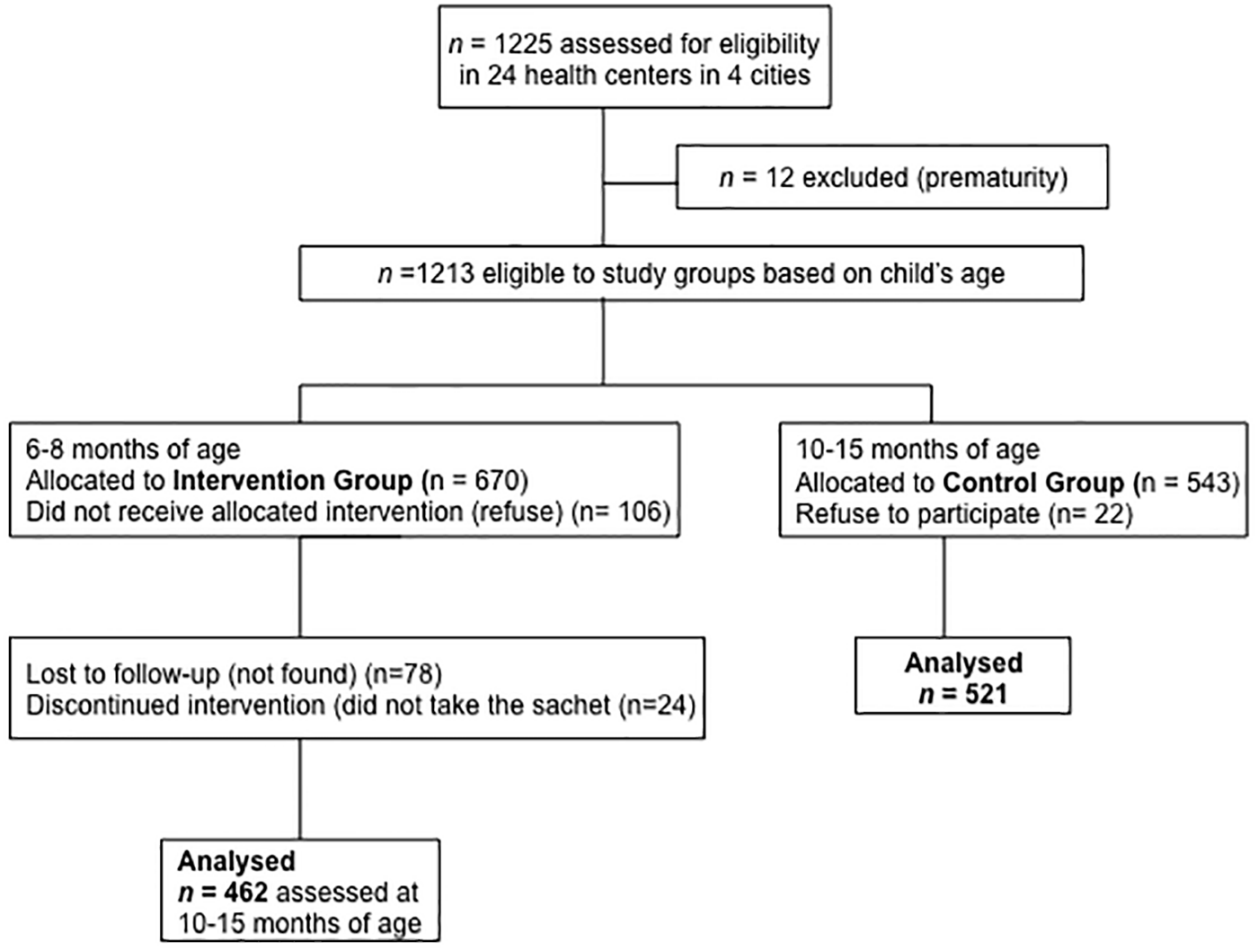
Flow of participants in the ENFAC study. CG, control group; ENFAC, *Estudo Nacional de Fortificação caseira da Alimentação Complementar*; IG, intervention group.

A total of 1225 children were recruited for the study, of whom 1213 were eligible (12 were excluded because of prematurity). Based on age, 543 children aged 10–15 months were selected as the CG. Of these, parents of 22 children (4.1%) declined participation. The remaining 521 caregivers (96% of the eligible children) completed the study interview and were scheduled for blood sampling. Among the 670 infants aged 6–8 months, parents of 108 (16.1%) declined; the remaining 564 were assigned to receive MNP from professionals at primary health study centers. Of these, 24 (4.3%) could not get the MNP with the health professionals, 78 (13.8%) were lost in the 4–6 months of follow-up due to address change, and the remaining 462 (81.9% of eligible infants) were included in this analysis as the IG (Fig 1). There was no significant difference in age and sex distribution among children lost to follow-up and children included in the analyses (*P>0*.*010)*.

The overall compliance reported by caregivers of the IG infants was 97%. Based on leftover sachets recorded at the end of the trial, 352 (76%) of the IG infants consumed at least 30 sachets; only 36% consumed all 60 sachets as recommended.

The general characteristics of the study participants are shown in Table 2. There were no significant differences between the study groups based in sex, birth weight, or race/ethnicity. However, IG children were on average 25 days younger than CG children. Although the mean years of maternal schooling was similar between groups (mean = 9.0 y; SD = 3.0 y), the proportion of mothers with less than 9 y of schooling was lower in the IG group (33.8%) than in CG (40.1%). Because of these differences, in addition to adjusting for health center and city in multilevel analysis, we further adjusted for child’s age and maternal schooling for comparisons between groups. The number of reported episodes of fever or wheezing in the prior15 days was statistically lower in the IG than in the CG (Table 2). No cases of current or past malaria infection were reported.

**Table 2:** Characteristics of the participants by ENFAC study groups^a^.

	CG (*n* = 521)	IG (*n* = 462)	*P*
Female Sex, *n* (%)	258 (49.5)	234 (50.7)	0.724
Birth weight (g), mean (SD)	3241.0 (493.2)	3280.5 (492.5)	0.213
Race/ethnicity:			0.685
Brown, *n* (%)	386 (74.4)	351 (76.8)	
White, *n* (%)	86 (16.5)	67 (14.7)	
Black, *n* (%)	32 (6.2)	29 (6.4)	
Maternal education <9 y, *n* (%)	204 (40.1)	153 (33.8)^2^	0.039
Weight (kg), mean (SD)	10.04 (1.3)	10.00 (1.4)	0.683
Height (cm), mean (SD)	76.11 (3.6)	75.76 (3.3)	0.117
HAZ, mean (SD)	-0.19 (1.14)	0.05 (1.15)	0.001
Breast feeding ≥ 6 months, *n* (%)	368 (71,2)	315 (68,6)	0.385

^a^Values are mean (SD) or percentages. Totals differ from the total number of study children due to missing values. Pearson χ^2^ and Student t tests were used to examine differences in proportions and in continuous variables between groups, respectively.

CG, control group; ENFAC Study, *Estudo Nacional de Fortificação caseira da Alimentação Complementar*; HAZ, Z-scores for length-for-age; IG, intervention group.

The crude prevalence of anemia was 38.1% lower in the IG than in the CG; ID was 19.9% lower, IDA was 51.4% lower, and vitamin A deficiency was 54.7% lower (*P* < 0.001, χ^2^ test; Table 3). The adjusted prevalence ratios showed a similar pattern for all these indicators, with the exception of ID, for which 95% CI of the adjusted PR was no longer significant. The concentrations of Hb, sTfR, folate, and beta-carotene were significantly higher in the IG compared with the CG. The proportion of vitamin E insufficiency was significantly lower in the IG than in the CG. Although the median PF values were not different between groups, the other indicators of short- (AGP> 1g/L) and long-term (CRP > 5mg/L) infection status were statistically different, with values 27% and 49% lower, respectively, in the IG.

**Table 3:** Prevalence of anemia and biochemical indicators by study groups at the end of the ENFAC Study^a^.

	CG, *n* = 521	IG, *n* = 462	*P*
Age (months), mean (SD)	13.5 (1.0)	12.7 (1.1)	< 0.001
Hemoglobin (g/L), mean (SD)	116.6 (13.5)	120.4 (10.6)	< 0.001
Anemia (Hemoglobin <110 g/L), *n* (%)	120 (23.1)	66 (14.3)	< 0.001
Anemia, adjusted PR (95% CI)^b^	1	0.63 (0.45, 0.88)	0.004
ID^c^, *n* (%)	188 (37.4)	128 (30.1)	0.020
ID^c^, adjusted PR (95% CI)^b^	1	0.81 (0.64, 1.04)	0.103
IDA^d^, *n* (%)	52 (10.3)	21 (4.9)	0.002
IDA^d^, adjusted PR (95% CI)^b^	1	0.45 (0.26, 0.77)	0.004
Plasma ferritin (μg/L), median (IQR)	19.9 (12.7; 33.5)	21.4 (13.0, 34.3)	0.688
sTfR (mg/L), median (IQR)	3.26 (1.23, 8.31)	2.87 (1.03, 5.21)	< 0.001
Vitamin A deficiency (<0.70μmol/L), *n* (%)	86 (17.4)	33 (7.9)	< 0.001
Vitamin A deficiency, adjusted PR (95% CI)^b^	1	0.45 (0.29, 0.69)	< 0.001
Vitamin E insufficiency (<11.6 μmol/L), *n*(%)	302 (61.5)	103 (24.9)	< 0.001
Vitamin E insufficiency, adjusted PR (95% CI)^b^	1	0.38 (0.30, 0.49)	< 0.001
Serum beta-carotene (μmol/L), median (IQR)	0.28 (0.14, 0.50)	0.36 (0.21, 0.60)	< 0.001
Folate, median (IQR)	39.66 (28.78, 55.29)	48.27 (34.44, 55.29)	< 0.001
AGP >1g/L, *n* (%)	183 (31.7)	116 (24.6)	0.001
AGP > 1 g/L, adjusted PR (95% CI)^b^	1	0.73 (0.55, 0.97)	0.030
CRP >5 mg/L, *n* (%)	79 (16.9)	33 (8.9)	< 0.001
CRP > 5 mg/L, adjusted PR (95% CI)^b^	1	0.512 (0.33, 0.80)	0.003

^a^Totals differ from the total number of study children due to missing values. AGP, α-1-acid glycoprotein; CG, control group; CRP, C-reactive protein; ENFAC, *Estudo Nacional de Fortificação caseira da Alimentação Complementar*; ID, iron deficiency; IDA, iron deficiency anemia; IG, intervention group; PR, prevalence ratio; sTfR, soluble transferrin receptor.

^b^*P* values were determined by testing the null hypothesis that each variable is equal between groups by using mixed-effects Poisson regression models, adjusted for health center, city, maternal education and child age (days).

^c^Defined with plasma ferritin < 12 ug/L or sTfR > 8.3 mg/L.

^d^Defined as Hb < 110 g/L with plasma ferritin < 12 ug/L or sTfR > 8.3 mg/L.

**Fig 2:**
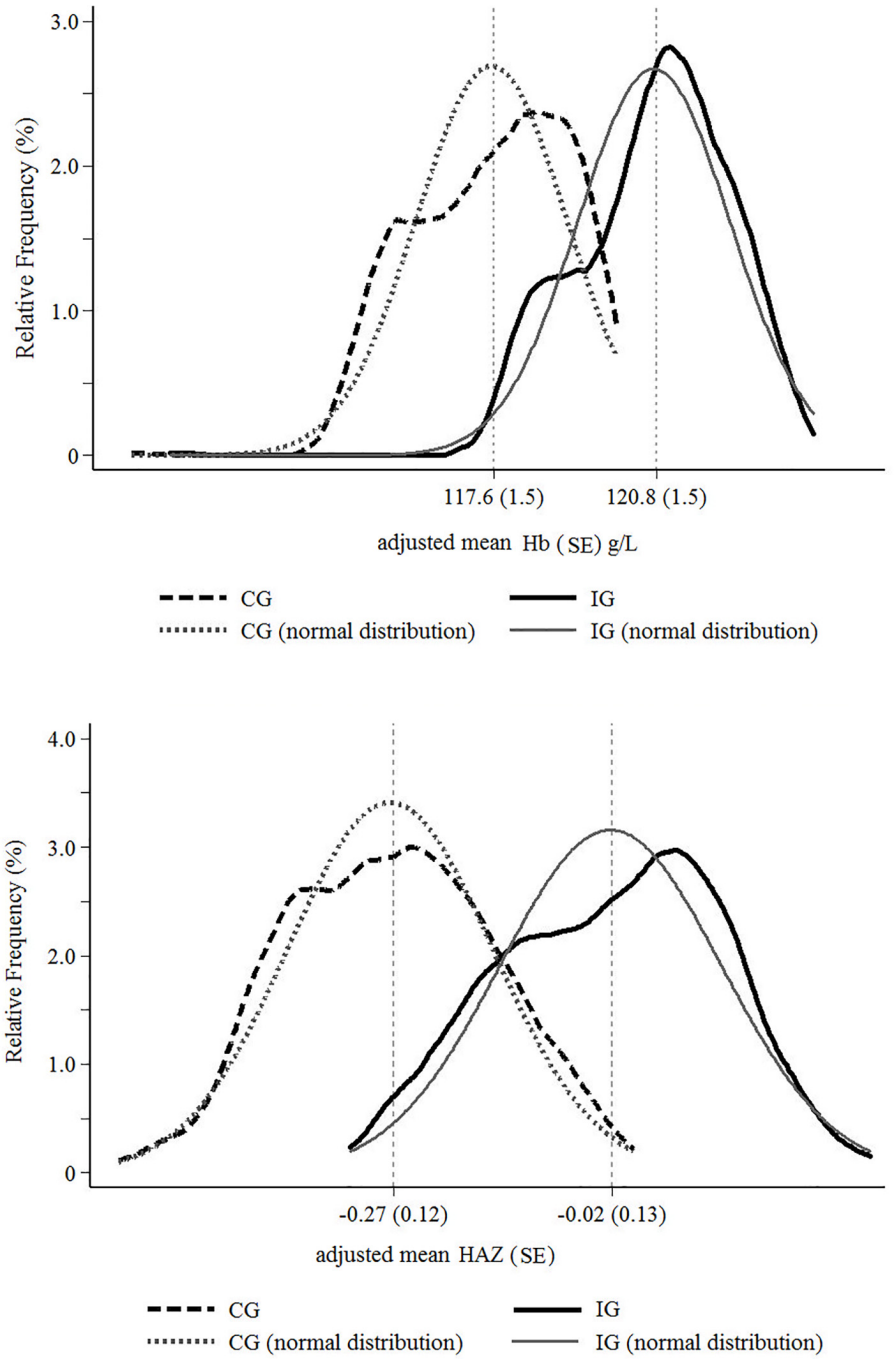
Relative frequency of hemoglobin (Hb in *A*) and Z-scores for length-for-age (HAZ in *B*) values adjusted for primary health center, city, child age and maternal schooling in multilevel linear regression analysis by ENFAC study groups. CG, control group; ENFAC, *Estudo Nacional de Fortificação caseira da Alimentação Complementar*; HAZ, lenght/height-for-age Z-score; Hb, hemoglobina; IG, intervention group.

The number (%) of stunted children was similar between study groups: 25 (4.9) and 19 (4.2) in CG and IG, respectively. The prevalence of folate deficiency was less than 1% in both groups: four children in CG and 2 in IG (results not shown). Fig 2 illustrates the Hb and HAZ distributions of the study groups using individual level data adjusted for health center, city, maternal schooling, and child age. Adjusted mean Hb was by 3.2 g/L higher in the IG (*P* < 0.001), which represents a shift to the right in the adjusted Hb distribution. This difference was even greater when considering only anemic children: the adjusted mean Hb for underlying density was 5.1 g/L higher in the anemic IG children compared with CG children. A similar pattern was found for the adjusted mean HAZ, with difference of 0.25 z-score, corresponding to a shift to the right in the adjusted HAZ distribution.

**Fig 3:**
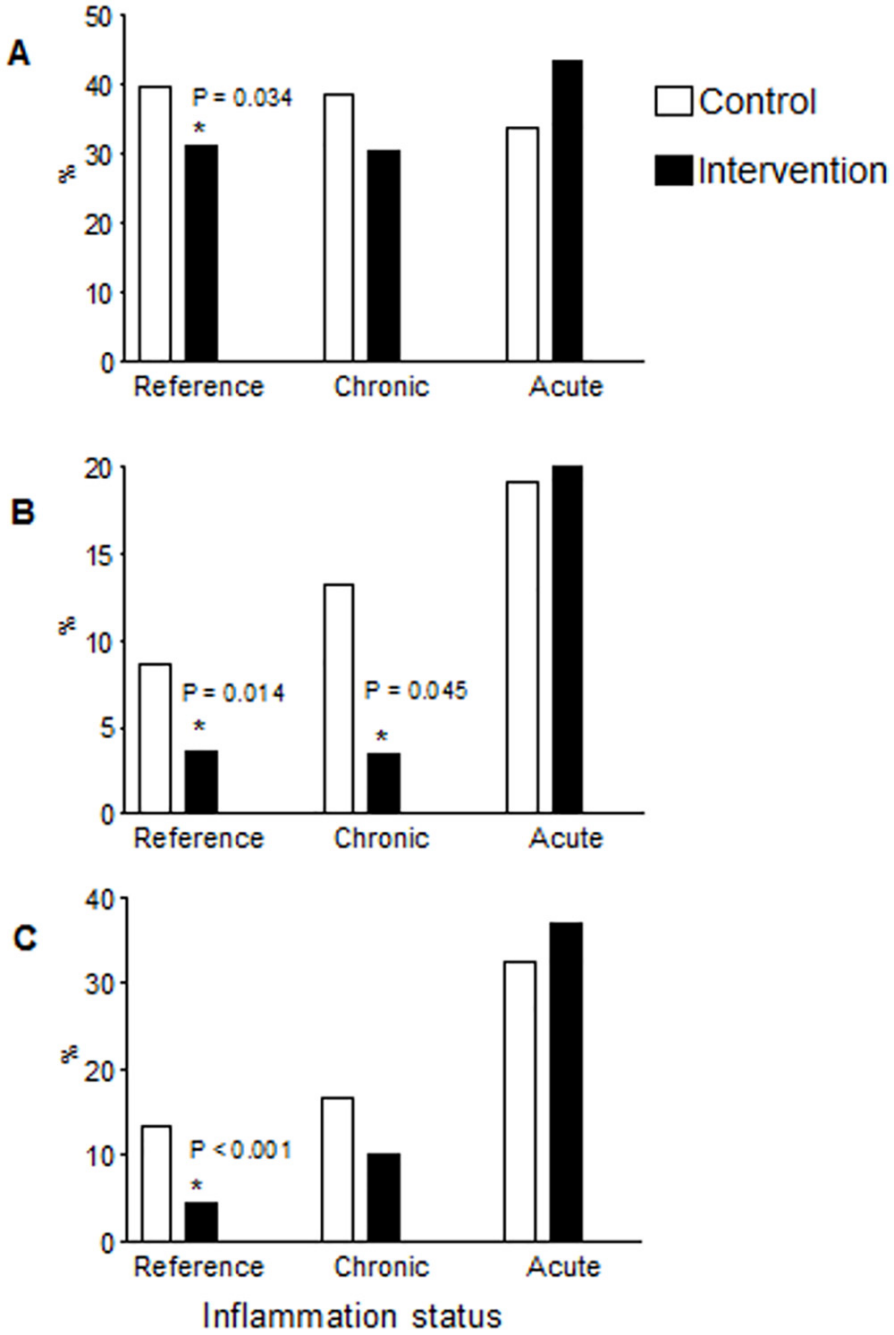
Frequency (%) of iron deficiency (*A*), iron deficiency anemia (*B)* and vitamin A deficiency (*C*) stratified by inflammation status using combined plasma values of CRP and AGP according to ENFAC study groups. Reference, CRP ≤ 5mg/L and AGP ≤ 1g/L, *n* = 302 (*A* and *B*) or 296 (*C*) in CG, and *n* = 275 (*A* and *B*) or 274 (*C*) in IG; Chronic, CRP ≤ 5mg/L and AGP> 1g/L, *n* = 83 (*A* and *B*) or 84 (*C*) in CG, and *n* = 59 (*A*, *B* or *C*) in IG; Acute, CRP >5 and AGP> 1g/L mg/L, *n* = 68 (*A* and *B*) or 65 (*C)* in CG, and *n* = 30 (*A* and *B*) or 27 (*C*) in IG. Totals differ from the total number of study children due to missing values for biochemical indicators. The symbol * indicates significant differences for the IG compared with CG (Pearson χ^2^ test). AGP, α-1-acid glycoprotein; CG, control group; CRP, C-reactive protein; ENFAC, *Estudo Nacional de Fortificação caseira da Alimentação Complementar;* IG, intervention group.

Because inflammation and infection may increase PF concentrations and decrease retinol concentrations, estimates of the effect of the intervention on the prevalence of ID, IDA, and VAD according to inflammation status are shown in Fig 3. The prevalence of ID, IDA, and VAD were significantly lower (~10%) in the IG infants in the absence of infection. Children with CRP ≥ 10 mg/L were included in this comparison (n = 42 in CG and n = 13 in IG children).

## Discussion

In this pragmatic trial, home fortification of complementary feeding with 60 sachets of MNP given on a flexible basis improved Hb concentrations and linear growth, and reduced anemia, iron and vitamin A deficiencies among Brazilian children aged 10–15 months. To our knowledge, this study is the first pragmatic trial with a large enough sample to have adequate power in a middle-income country. Since the use of the MNP requires its addition to semi-solid foods (such as mashed fruits and vegetables), in this study the intervention with MNP optimized the effects of primary healthcare strategies to promote healthy complementary feeding, further improving the benefits of the home fortification.

The reduction in anemia observed in this study was similar to the findings of other studies on the effectiveness of home fortification with MNP. In 2009, a Cochrane systematic review suggested that home fortification with MNP is as effective as iron supplementation in treating anemia with better acceptance resulting from reduced side effects [14]. The second Cochrane systematic review assessed the effects on health outcomes of home fortification with MNP of foods consumed by children under 2 years of age [15]. That review included eight trials conducted in low-income countries in Asia, Africa, and the Caribbean. The interventions lasted for 2–12 months; only one study evaluated the use of MNP on a flexible basis (but no more than one sachet per day). The overall quality of the evidence for ID was considered high, whereas evidence was moderate for anemia, Hb concentration, iron status, and growth. Home fortification of foods with MNP reduced anemia by 31% (average RR 0.69, 95% CI 0.60–0.78) and ID by 51% (average RR 0.49, 95% CI 0.35–0.67) when compared with no intervention or placebo. However, fortification had no effect on HAZ, and diarrhea was reported in only five trials.

Recently, clinical trial studies have been published about the effects of MNP on other nutritional outcomes [26–29]. Variations in the number of micronutrients used in the sachets (ranging from 3 to 13 nutrients), duration of the intervention (from 6 to 24 months), age group (ranging from 6 to 48 months) and nutritional profile of the target population could explain some findings. While home fortification with MNP did reduce the prevalence of anemia in those studies, it did not result in significant improvement in anthropometric outcomes, although favorable trends were found. One of these studies, a large cluster-randomized trial in urban and rural sites in Pakistan [27], found a reduction in IDA, with a small effect on growth. MNPs were given daily between 6 and 18 months of age; the prevalence of anemia and stunting at baseline ranged from 72–92% and 23–30%, respectively. An increased proportion of days with diarrhea was reported in the intervention group compared to control. Because diarrhea is well recognized as a potential adverse effect of iron supplementation in malnourished children, the increased diarrhea incidence could explain the reduced growth benefits of MNP [30]. In another large cluster randomized trial of young children in a rural community setting in Ghana [29], malaria incidence was significantly lower in the group using MNP with iron compared with the iron-free MNP group; no differences in anthropometric measures were reported. In our study, the following possible factors could contribute to the improvement observed in the HAZ distribution related to the use of MNP: reported frequency of diarrhea was similar in the IG and CG children, and reported episodes of fever and wheezing in the prior 15 days and levels of inflammatory markers were statistically lower in the IG than in the CG. These results could be related to a better nutritional status observed in IG children. However, caution should be considered in this interpretation as our study design did not provide follow-up information for unbiased comparison with the CG children.

Another important finding from our study is the reduced prevalence of VAD in the IG children. Additional information on the later stages of inflammation could discern the effect of recurrent infection on iron and vitamin A status [31]. The prevalence of ID, IDA, and VAD were significantly (10%) lower in the IG infants in the absence of infection. In contrast to our findings, previous studies [26, 32–33] did not find significant impact of the MNP on vitamin A status, partially attributable to the insensitivity of indicators used to assess vitamin A status, and the relatively low prevalence of vitamin A deficiency in the population without inflammation [33].

Some limitations of our study should be noted. While the general characteristics of the study children were similar between groups, this pragmatic controlled clinical trial design does not allow randomization and blinding. Nevertheless, the results were obtained in the routine situation, providing valuable information about the effectiveness of the use of MNP through primary healthcare in different regions in Brazil. Although we found that MNP was effective in reducing anemia, and improving growth and micronutrient status, logistic constraints prevented measurement of other biochemical indicators, including zinc status, and detailed dietary intake information was not collected at all study centers. Thus, we have no information on whether the quality of complementary feeding improved over the course of the study. However, IG participants had higher serum concentrations of beta-carotene and vitamin E, which are good biomarkers of habitual consumption of fruits and vegetables, suggesting that our results are probably associated in part with improved quality of complementary feeding following the recommendations for use of MNP delivered through primary healthcare.

In conclusion, our trial confirmed that the home fortification with MNP given once daily for 2–3 months and provided through primary healthcare can reduce anemia and improve growth, micronutrient status, and morbidity status among Brazilian children.

Our findings support the conclusions of previous studies that MNP interventions should be integrated with health care and education, starting at 6–8 months of age with child’s diet transition from breast feeding to complementary feeding, for improvements in linear growth and micronutrient status.

## Supporting Information

S1 Text Trial study protocol. (PDF)

S2 Text TREND statement checklist for reporting non-randomized trials. (PDF)
