# Correction: Effect of Providing Multiple Micronutrients in Powder through Primary Healthcare on Anemia in Young Brazilian Children: A Multicentre Pragmatic Controlled Trial

**DOI:** 10.1371/journal.pone.0156194

**Published:** 2016-05-18

**Authors:** Marly A. Cardoso, Rosangela A. Augusto, Gisele A. Bortolini, Cristieli S. M. Oliveira, Daniela C. Tietzman, Leopoldina A. S. Sequeira, Maria Claret C. M. Hadler, Maria do Rosario G. Peixoto, Pascoal T. Muniz, Márcia R. Vitolo, Pedro I. C. Lira, Patrícia C. Jaime, ENFAC Working Group

In Fig 2, the graphs should be labelled “A” and “B” to indicate separate panels. Please see the corrected Fig 2 here.

**Fig 2:**
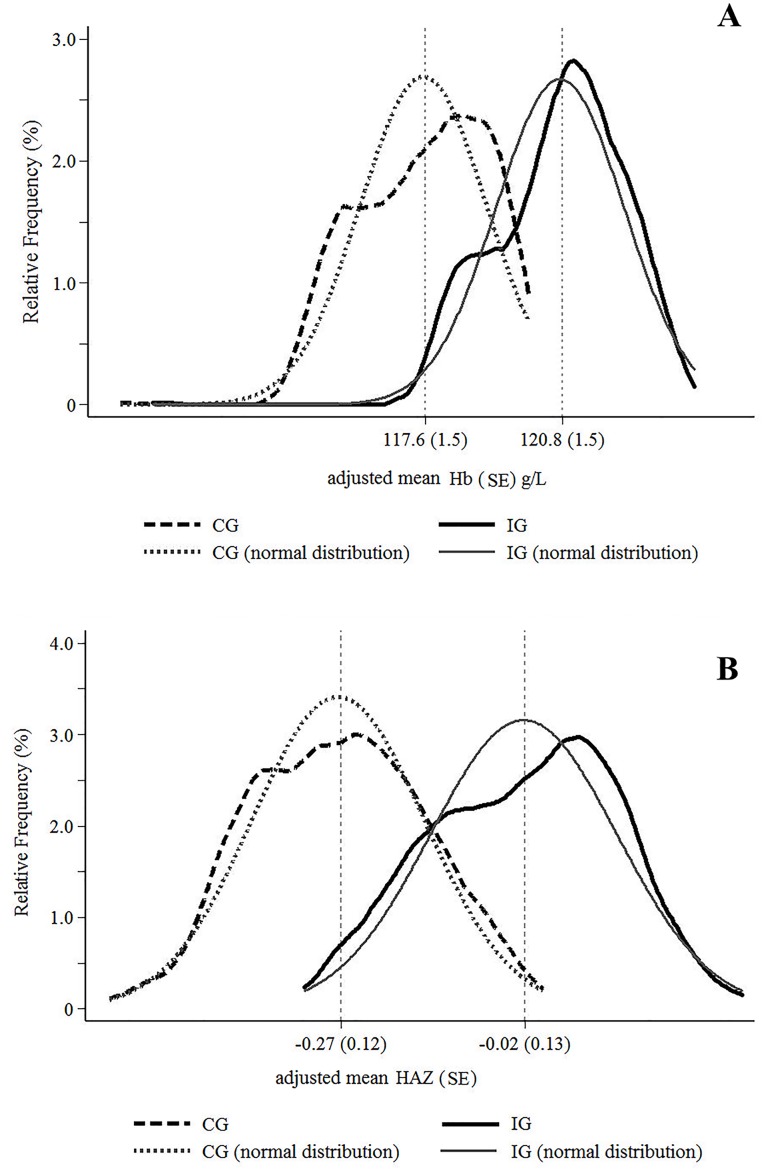
Relative frequency of hemoglobin (Hb in A) and Z-scores for length-for-age (HAZ in B) values adjusted for primary health center, city, child age and maternal schooling in multilevel linear regression analysis by ENFAC study groups. CG, control group; ENFAC, Estudo Nacional de Fortificação caseira da Alimentação Complementar; HAZ, lenght/height-for-age Z-score; Hb, hemoglobina; IG, intervention group.
