# Supplementary material for: Effect of Providing Multiple Micronutrients in Powder through Primary Healthcare on Anemia in Young Brazilian Children: A Multicentre Pragmatic Controlled Trial
Source: PLoS One. 2016 Mar 14;11(3):e0151097. doi: 10.1371/journal.pone.0151097 (PMC4790963; doi:10.1371/journal.pone.0151097)
Supplement: S1 Text — (PDF) [file pone.0151097.s001.pdf]

Supporting Information File

Trial study protocol at www.ensaiosclinicos.gov.br

RBR-5ktv6b
Effectiveness of home fortification with vitamins and minerals for the prevention of iron deficiency and anemia in infants younger than 1 year of age: a multi-center study of Brazilian cities.

Registration Date: April 3, 2012, 1:42 p.m.

Last Update: Sept. 11, 2013, 10:10 a.m.

Study Type:

Intervention Study

Scientific Title:

Efetividade da fortificação caseira com vitaminas e minerais na prevenção da deficiência de ferro e anemia em crianças menores de um ano: estudo multicêntrico em cidades brasileiras.

Effectiveness of home fortification with vitamins and minerals for the prevention of iron deficiency and anemia in infants younger than 1 year of age: a multi-center study of Brazilian cities.

Trial Identification

- **UTN Number:** U1111-1129-7089

- **Public Title:**

  Fortificação caseira da alimentação complementar no Brasil: ensaio pragmático multicêntrico

  Home fortification of complementary feeding in Brazil: a multicenter pragmatic trial

- **Scientific Acronym:**

  ENFAC: Estudo Nacional de Fortificação caseira da Alimentação Complementar

ENFAC: Brazilian study for home fortification of complementary feeding

- **Public Acronym:**

ENFAC: Estudo Nacional de Fortificação caseira da Alimentação Complementar

ENFAC: Brazilian study for home fortification of complementary feeding

- **Secondary Identifying Numbers:**
  - **2291/2012**
    Issuing Authority: Comitê de Ética em Pesquis da Faculdade de Saúde Pública da Universidade de São Paulo

Sponsors

- **Primary Sponsor:** Ministério da Saúde

- **Secondary Sponsors:**
  - **Institution:** CNPq

- **Source(s) of Monetary or Material Support:**
  - **Institution:** Ministério da Saúde
  - **Institution:** UNICEF

Health Conditions

- **Health Condition(s) or Problem(s):**

Anemia Ferropriva

Anemia, Iron-Deficiency

- **General Descriptors for Health Condition(s):**

C18: Doenças nutricionais e metabólicas

C18: Enfermedades nutricionales y metabólicas

C18: Nutritional and metabolic diseases

- **Specific Descriptors for Health Condition(s):**

C15.378.071.196.300: Anemia Ferropriva

C15.378.071.196.300: Anemia Ferropénica

C15.378.071.196.300: Anemia, Iron-Deficiency

Interventions

- Intervention Code(s)
  - **Dietary supplement**

- **Interventions:**

PT-BR

Grupo experimental: 675 crianças de 6-8 meses acompanhadas na atenção básica à saúde (modelos tradicional e programa de saúde da família) receberão 60 sachês de 1 g cada de polivitamínico e mineral, para ser administrado 1 vez por dia com alimentos semi-sólidos (papas de fruta ou papas salgadas) na hora de servir, durante 60 dias. Composição do sachê (1g) polivitamínico e mineral: Ferro 10 mg, Zinco 4,1 mg, Ácido fólico 150 µg, Vitamina A 400 µg RE, Vitamina C 30 mg, Vitamina D3 5 µg, Vitamina E 5 mg TE, Vitamina B1 0,5 mg, Vitamina B2 0,5 mg, Vitamina B6 0,5 mg, Vitamina B12 0,9 µg, Niacina 6 mg, Cobre 0,56 mg, Iodo 90 µg, Selênio 17 µg.

Grupo controle: 675 crianças de 12 a 14 meses de idade acompanhadas na atenção básica à saúde (modelos tradicional e programa de saúde da família) em atendimento de acordo com a rotina de puericultura vigente com programa de suplementação com sulfato ferroso.

EN

Intervention group: 675 infants aged 6-8 months at recruitment from basic health care (traditional and health Family programs)will receive 60 multiple micronutrient powder (MNP)to be given once a day with semi-solid foods (fruit puree or salty meals) during 60 days. The composition of each sachet (1g): Iron 10 mg, Zinc 4,1 mg, Folate 150 µg, Vitamin A 400 µg RE, Vitamin C 30 mg, Vitamin D3 5 µg,Vitamin E 5 mg TE, Vitamin B1 0,5 mg, Vitamin B2 0,5 mg, Vitamin B6 0,5 mg, Vitamin B12 0,9 µg, Niacin 6 mg, Cupper 0,56 mg, Iodine 90 µg, Selenium 17 µg.

Control group: 675 infants aged 12-14 months attending at the current usual basic health care (traditional and health Family programs)attending at the current routine care with ferrous sulfate supplementation.

- **Descriptor for Intervention(s):**

SP6.051.227: Suplementação Alimentar

SP6.051.227: Alimentación Suplementaria

Recruitment

- **Recruitment Status:** Recruitment completed

- <u>**Recruitment Country**</u>
  - Brazil

- **Planned Date of First Enrollment:** 2012-06-13

- **Planned Date of Last Enrollment:** 2013-01-04

-

Target Sample Size:	Gender (inclusion sex):	Inclusion Minimum Age:	Inclusion Maximum Age:
1350	-	6 M	15 M

- **Inclusion Criteria:**

Grupo intervenção: crianças entre 6 e 8 meses de idade no recrutamento (6 meses antes da medida de desfecho)

Grupo controle: crianças entre 12 e 14 meses para medida de desfecho.

Intervention group: infants between 6 and 8 months at the enrollment(6 months before outcome measures)

Control group: infants between 12 and 14 months at outcome measures

- **Exclusion Criteria:**

Casos referidos de malária, positividade para HIV, tuberculose, hemoglobinopatias e estar em tratamento para anemia.

Reported cases of malaria, positive for HIV, tuberculosis, hemoglobinopathies, and in treatment for anemia.

Study Type

- **Study Design:**

Ensaio clínico de prevenção, controlado não-randomizado, paralelo, aberto, com dois braços, prospectivo, de fase 3.

Prevention Clinical trial, non-randomized controlled, parallel, open, 2 arms, prospective, fase 3.

Expanded access program	Study Purpose	Intervention Assignment	Number of arms	Masking type	Allocation type	Study Phase
False	Prevention	Parallel	2	Open	Non-randomized-controlled	3

Outcomes

- **Primary Outcomes:**

  Diferença média de hemoglobina sanguínea de pelo menos 6 g/L

  A mean diference in the blood hemoglobin of at least 6 g/L

- **Secondary Outcomes:**

  Redução da prevalência de anemia e deficiência de ferro.

  Decreased prevalence of anemia and iron deficiency.

Contacts

- <u>Contacts for Public Queries</u>
  - **Full Name:** Marly Augusto Cardoso
    - **Address:** Av Dr Arnaldo 715
    - **City:** São Paulo / Brazil
    - **Zip Code:** 01246--904
  - **Telephone:** +5-5-11--30617705
  - **E-mail:** marlyac@usp.br
  - **Affiliation:** University of Sao Paulo
